# Immune-Modulatory Effects upon Oral Application of Cumin-Essential-Oil to Mice Suffering from Acute Campylobacteriosis

**DOI:** 10.3390/pathogens10070818

**Published:** 2021-06-29

**Authors:** Soraya Mousavi, Dennis Weschka, Stefan Bereswill, Markus M. Heimesaat

**Affiliations:** Gastrointestinal Microbiology Research Group, Institute of Microbiology, Infectious Diseases and Immunology, Charité-Universitätsmedizin Berlin, Corporate Member of Freie Universität Berlin, Humboldt-Universität zu Berlin, and Berlin Institute of Health, 12203 Berlin, Germany; soraya.mousavi@charite.de (S.M.); dennis.weschka@charite.de (D.W.); stefan.bereswill@charite.de (S.B.)

**Keywords:** cumin-essential-oil, cuminaldehyde, *Campylobacter jejuni*, enteropathogenic infection, immune-modulatory effects, secondary abiotic IL-10^-/-^ mice, experimental campylobacteriosis model, host–pathogen interaction, preclinical intervention study, natural antibiotics-independent compounds

## Abstract

Human campylobacteriosis, commonly caused by *Campylobacter jejuni,* is a food-borne infection with rising prevalence causing significant health and socioeconomic burdens worldwide. Given the threat from emerging antimicrobial resistances, the treatment of infectious diseases with antibiotics-independent natural compounds is utmost appreciated. Since the health-beneficial effects of cumin-essential-oil (EO) have been known for centuries, its potential anti-pathogenic and immune-modulatory effects during acute experimental campylobacteriosis were addressed in the present study. Therefore, *C. jejuni*-challenged secondary abiotic IL-10^-/-^ mice were treated perorally with either cumin-EO or placebo starting on day 2 post-infection. On day 6 post-infection, cumin-EO treated mice harbored lower ileal pathogen numbers and exhibited a better clinical outcome when compared to placebo controls. Furthermore, cumin-EO treatment alleviated enteropathogen-induced apoptotic cell responses in colonic epithelia. Whereas, on day 6 post-infection, a dampened secretion of pro-inflammatory mediators, including nitric oxide and IFN-γ to basal levels, could be assessed in mesenteric lymph nodes of cumin-EO treated mice, systemic MCP-1 concentrations were elevated in placebo counterparts only. In conclusion, our preclinical intervention study provides first evidence for promising immune-modulatory effects of cumin-EO in the combat of human campylobacteriosis. Future studies should address antimicrobial and immune-modulatory effects of natural compounds as adjunct antibiotics-independent treatment option for infectious diseases.

## 1. Introduction

*Campylobacter* species belong to the commensal gut microbiota of animal species used for food production, such as chicken, turkey and other poultry [1,2]. Ingestion of meat products or surface waters contaminated with *Campylobacter* bacteria is the main source for human campylobacteriosis [3,4]. *Campylobacter jejuni* constitute the most common foodborne enteropathogens in the European Union, exceeding *Salmonella* infections with progressively increasing case numbers [5]. The highly motile Gram-negative *C. jejuni* bacteria bypass the viscous intestinal mucus layer, cross the epithelia, and interact with mucosal and lamina propria cells, leading to the recruitment of innate immune cells, such as neutrophils, dendritic cells, macrophages, and monocytes [6]. The interaction between the Toll-like receptor-4 (TLR-4) expressed by these immune cell subsets with the surface lipooligosaccharide (LOS) derived from the *C. jejuni* cell wall leads to a massive pro-inflammatory mediator response, inducing apoptosis and ulcerations in the intestinal epithelia, followed by impaired epithelial barrier function and malabsorption [6,7]. The classical clinical signs of human campylobacteriosis are characterized by diarrhea with mucous, inflammatory or even bloody discharge, abdominal cramping, and fever [8,9]. In most cases, the disease course is self-limiting, resolving within approximately 7 to 14 days post-infection (p.i.), and requires (if at all) a symptomatic regimen consisting of antipyretics and substitution of fluids and electrolytes. Antibiotics may be indicated in severe cases, particularly in immune-compromised patients [8,10,11,12]. Guillain–Barré syndrome, Miller Fisher syndrome, irritable bowel disease, inflammatory bowel diseases, and coeliac disease can develop as post-infectious morbidities in relatively rare instances after weeks or even months [2,7,8,12,13,14].

Experimental animals, such as conventional laboratory mice, are highly resistant against *C. jejuni* infection, which also holds true for TLR-4 ligands derived from the Gram-negative bacteria, including carbohydrate structures such as LOS and lipopolysaccharides (LPS) [15]. However, the depletion of the complex murine gastrointestinal microbiota by antibiotic compounds has been shown to sufficiently override the physiological colonization resistance against the enteropathogens and thus, facilitate the establishment of *C. jejuni* in the murine gastrointestinal tract [16]. Within six days post *C. jejuni* challenge, secondary abiotic mice lacking the *interleukin-10* (IL-10) gene (IL-10^-/-^ mice) suffer from severe campylobacteriosis. The disease is characterized by bloody diarrhea and wasting symptoms and presents with acute enterocolitis, intestinal apoptosis, and pronounced pro-inflammatory immune responses that are not limited to the intestinal tract but also affect extra-intestinal and even systemic tissue sites [6,17]. In our recent preclinical intervention studies, secondary abiotic IL-10^-/-^ mice have been successfully applied to survey anti-microbial and immune-modulatory properties of distinct molecules such as vitamin C [18]; vitamin D [19]; carvacrol [20]; urolithin-A [21]; resveratrol [22]; essential oils (EO), including cardamom-EO [23] and clove-EO [24]; as well as the neuropeptides PACAP [25] and NAP [26] in acute campylobacteriosis. Furthermore, given the emergence of rising antimicrobial resistance of bacterial pathogens also affecting *Campylobacter* species, we are searching for natural compounds with non-toxic, anti-pathogenic, and/or immune-modulatory properties that might be promising options for the treatment and/or even prophylaxis of campylobacteriosis.

Cumin (*Cuminum cyminum* Linn.) is an aromatic medical plant belonging to the *Apiaceae* family and is native in Middle Eastern countries [27,28]. The health-beneficial anti-microbial, anti-inflammatory, and anti-oxidant effects of this natural compound have been known for a long time. In traditional medicine, cumin and derivatives have, therefore, been used to treat a wide spectrum of diseases, such as gastrointestinal morbidities including infectious diarrhea [27,29,30,31,32]. The major bioactive constituents of cumin-EO are cuminaldehyde, eugenol, α-pinene, cymene, and terpenoids [29,33]. Cumin-EO has antimicrobial activity against various bacteria, such as *Staphylococcus aureus*, *Staphylococcus epidermidis*, *Staphylococcus haemolyticus*, *Klebsiella pneumoniae*, *Propionibacterium acnes*, and *Corynebacterium diphtheriae*, and against fungi such as *Candida albicans* [34,35,36]. An in vitro study revealed an increased permeability of the cell membrane of *Campylobacter* species, including *C. jejuni* and *C. coli*, upon co-incubation with cumin-EO [37]. The exact molecular mechanisms underlying the cumin-EO mediated toxicity against *C. jejuni* have not been examined further, however. Additionally, in vivo data of the beneficial effects exerted by cumin in *C. jejuni* infection are completely missing. Given our recent finding that the eugenol containing clove oil was highly effective in murine campylobacteriosis [24], this encouraged us to perform a preclinical intervention study regarding the anti-pathogenic and immune-modulatory effects of cumin-EO during acute *C. jejuni* infection applied to secondary abiotic IL-10^-/-^ mice, as presented here.

## 2. Material and Methods

### 2.1. Experimental Animals and Husbandry

The breeding and keeping of the IL-10^-/-^ mice (C57BL/6j background) took place in the Forschungsinstitute für Experimentelle Medizin, Charité—Universitätsmedizin Berlin, Germany. The animals were housed under standard conditions (i.e., 22–24 °C room temperature, 55 ± 15% humidity, 12 h light/12 h dark cycle). The animals were kept in cages (maximum of 3–4 mice per cage) including filter tops within an experimental semi-barrier, supplied with drinking water (autoclaved tap water, ad libitum) and food pellets (ssniff R/M-H, V1534-300, Sniff, Soest, Germany). In order to deplete the commensal gut microbiota, 3-week-old animals were transferred into sterile cages and received a broad-spectrum antibiotic solution via the drinking water (ad libitum) for 8 weeks (Supplementary Table S1) as described earlier [16]. To avoid contaminations, the secondary abiotic IL-10^-/-^ mice were kept and handled under strict aseptic conditions. In order to ensure the washout of the antibiotics, the animals received autoclaved tap water three days before infection with *C. jejuni*.

### 2.2. Campylobacter jejuni Infection and Gastrointestinal Pathogen Loads

On days 0 and 1, 4-month-old secondary abiotic IL-10^-/-^ mice (sex-matched littermates; balanced gender ratio) were infected perorally with 10^9^ colony forming units (CFU) of the *C. jejuni* strain 81–176 grown on Columbia agar (with 5% sheep blood) and selective karmali agar plates (both from Oxoid, Wesel, Germany). Therefore, bacterial colonies were harvested with a sterile swab from respective agar plates after 48-h incubation under microaerophilic conditions and transferred to sterile phosphate-buffered saline (PBS, Thermo Fisher Scientific, Waltham, MA, USA). Immediately thereafter, mice received 0.3 mL of the bacterial suspension by gavage.

As described earlier, the daily pathogen loads were determined in fecal samples after *C. jejuni* infection and upon necropsy in luminal samples from the gastrointestinal tract (stomach, duodenum, ileum, and colon) by culture [16,38]. Briefly, serial dilutions of each sample (in sterile PBS, Thermo Fisher Scientific, Waltham, MA, USA) were plated onto karmali agar and incubated under microaerophilic conditions for at least 48 h and at 37 °C. The detection limit of viable pathogens was 100 CFU per g (CFU/g).

### 2.3. Clinical Outcome

The daily clinical outcomes of each mouse were quantitated by using a cumulative clinical score (Supplementary Table S2), as described previously [39].

### 2.4. Cumin-EO Treatment and Cohort Sizes

Starting on day 2 p.i and lasting until the end of the observation period, mice were treated with cumin-seed-EO (Sigma-Aldrich, Munich, Germany) via the drinking water. Cumin-EO was dissolved in sterile PBS with 0.05% carboxymethylcellulose, added to autoclaved tap water to a final concentration of 1 mg/mL (ad libitum). Assuming a daily drinking volume of approximately 5 mL and a mean body weight of 25 g, the daily cumin-EO dose applied to the mice of the verum group was 200 mg/kg body weight. Mice from the placebo cohort received the solution without the verum instead. Furthermore, naive mice were included as non-treated and uninfected controls. The four independent intervention studies were performed with the following cohort sizes: Naive cohort (4, 4, 4, 4); placebo treated and infected cohort (8, 7, 7, 7); cumin-EO treated and infected cohort (5, 5, 5, 4).

### 2.5. Sampling

On day 6 p.i., mice were sacrificed by CO_2_ asphyxiation. For serum cytokine measurements, cardiac blood samples were collected from each mouse. Ex vivo biopsies from mesenteric lymph nodes (MLN) and the colon, as well as luminal samples from the gastrointestinal tract, were derived under aseptic conditions. For further microbiological and immune-histopathological analyses, large intestinal biopsies were sampled in parallel.

### 2.6. Histopathological Analyses

For histopathological analyses, colonic ex vivo biopsies were fixed immediately in 5% formalin and embedded in paraffin. The sections (5 µm) stained with hematoxylin and eosin (H&E) were examined for histopathological changes in the large intestine by light microscopy (100-fold magnification). These changes were assessed quantitatively using histopathological ratings (Supplementary Table S3), as stated recently [40].

### 2.7. In Situ Immunohistochemistry

Quantitative in situ immunohistochemical analyses were performed on the colonic ex vivo biopsies (fixed immediately in 5% formalin and embedded in paraffin) to detect apoptotic epithelial cells, macrophages and monocytes, T lymphocytes, regulatory T cells, and B lymphocytes (Supplementary Table S4), as stated earlier [41,42]. The mean number of respective positively stained cells in each sample was determined within six high power fields (HPF, 0.287 mm^2^, 400-fold magnification) by a blinded independent investigator applying light microscopy.

### 2.8. Pro-Inflammatory Mediators

Intestinal ex vivo biopsies collected from the colon (longitudinally cut strips of approximately 1 cm^2^, washed in PBS) and from MLN (3 nodes) were incubated for 18 h at 37 °C in 24-flat-bottom-well culture plates (Thermo Fisher Scientific, Waltham, MA, USA) containing 500 mL serum-free RPMI 1640 medium (Thermo Fisher Scientific, Waltham, MA, USA) supplemented with penicillin (100 µg/mL) and streptomycin (100 µg/mL; Biochrom, Berlin, Germany). After the incubation, supernatants and serum samples were tested for interferon-γ (IFN-γ) and monocyte chemoattractant protein-1 (MCP-1) by the mouse inflammation cytometric bead assay (CBA; BD Biosciences, Heidelberg, Germany) in a BD FACSCanto II flow cytometer (BD Biosciences, Heidelberg, Germany). Nitric oxide concentrations were determined by the Griess reaction, as stated elsewhere [43,44].

### 2.9. Statistical Analyses

GraphPad Prism (version 8; San Diego, CA, USA) was used for the calculation of medians and significance levels. The normal distribution of the data was determined by the Anderson–Darling test. For not normally distributed data the Mann–Whitney test (for pairwise comparisons) and Kruskal–Wallis test with Dunn’s post-correction (for multiple comparisons) were applied. The one-way ANOVA with Tukey post-correction was performed on normally distributed data for multiple comparisons. Two-sided probability (*p*) values ≤ 0.05 were considered significant. Data were pooled from four independent experiments.

## 3. Results

### 3.1. Cumin-EO Treatment and Gastrointestinal C. jejuni Colonization in Secondary Abiotic IL-10^-/-^ Mice

**Figure 1:**
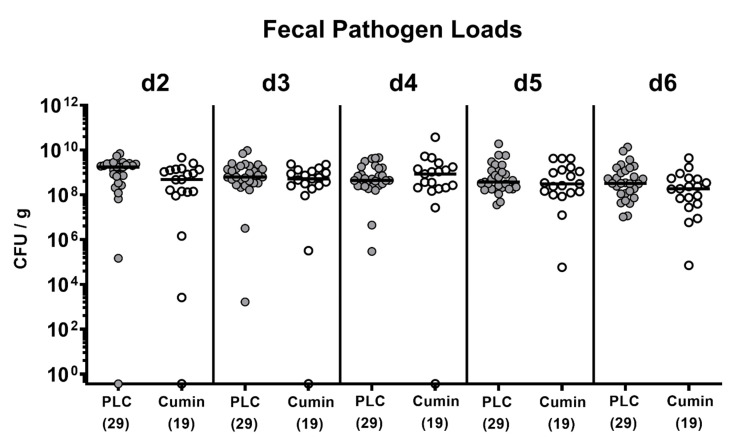
Kinetic analysis of fecal *C. jejuni* densities in infected IL-10^-/-^ mice during cumin-EO treatment. Secondary abiotic IL-10^-/-^ mice were infected with *C. jejuni* strain 81–176 on day (d) 0 and d1 by gavage and received either placebo (grey circles) or cumin-EO (white circles) via the drinking water from d2 until d6 post-infection. Medians (black bars) of *C. jejuni* colony forming units per gram of feces sample (CFU/g) determined by culture are shown. The numbers of analyzed mice (in parentheses) are given. Data were pooled from four independent experiments.

10^9^ viable *C. jejuni* strain 81–176 cells were applied to secondary abiotic IL-10^-/-^ mice on days 0 and 1 by gavage. On day 2 p.i., treatment with cumin-EO or placebo was initiated and continued until the end of the study (i.e., day 6 p.i.) via the drinking water. The cultural analyses of the fecal pathogen densities over time revealed comparably high median *C. jejuni* loads of 10^9^ colony forming units per g (CFU/g) feces derived from mice of both cohorts as early as 2 days p.i. that did not change thereafter (Figure 1).

**Figure 2:**
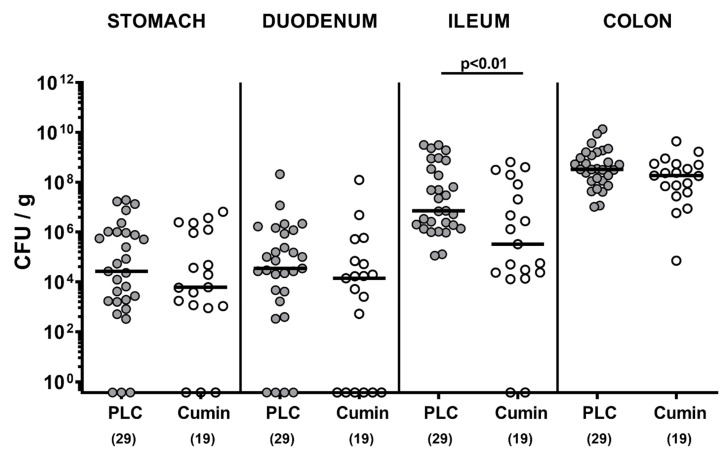
Effects of cumin-EO treatment on gastrointestinal pathogen loads. *C. jejuni* infected secondary abiotic IL-10^-/-^ mice received either placebo (PLC, grey circles) or cumin-EO (white circles) from d2 until d6 post-infection. Gastrointestinal pathogen numbers (colony forming units per gram, CFU/g) were determined on d6 post-infection by culture. Medians (black bars) and significance levels (*p* values) were analyzed with data pooled from four independent experiments using the Mann–Whitney U-test. The numbers of included mice are given in parentheses.

Interestingly, in a single mouse from the placebo and cumin-EO groups, the fecal *C. jejuni* numbers were below the detection limit during the early course of infection (i.e., on day 2 p.i. and between days 2 and 4 p.i., respectively). On the day of sacrifice at day 6 p.i., we performed a comparative survey of the pathogen loads along the gastrointestinal tract. Interestingly, our cultural analyses revealed approximately 1.5 log orders of magnitude lower *C. jejuni* cell numbers in the distal small intestines (i.e., the terminal ileum) of cumin-EO as compared to the placebo treated mice (*p* < 0.01), whereas in the stomach, the duodenum and the colon *C. jejuni* were comparably high in either cohort (not significant (n.s.); Figure 2). Hence, cumin-EO treatment resulted in lower *C. jejuni* loads in the ileum as compared to placebo.

### 3.2. Clinical outcome Following Cumin-EO Treatment of C. jejuni infected IL-10^-/-^ Mice

**Figure 3:**
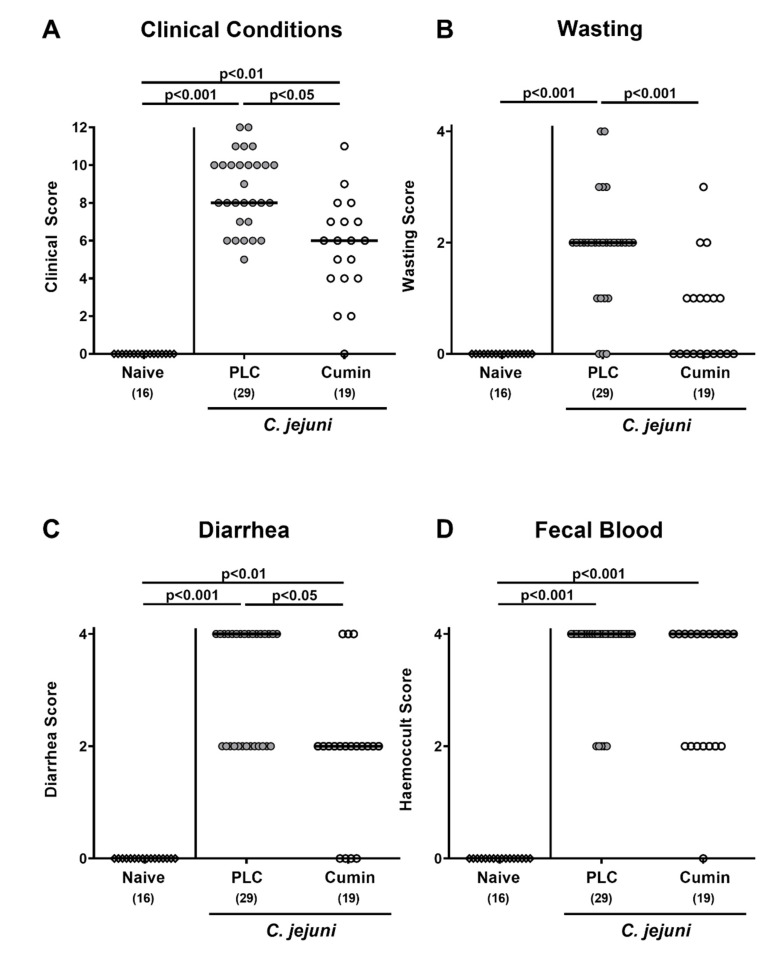
Effects of cumin-EO treatment on the clinical outcome of *C. jejuni* infection in IL-10^-/-^ mice. *C. jejuni* infected secondary abiotic IL-10^-/-^ mice received either placebo (PLC, grey circles) or cumin-EO (white circles) from d2 until d6 post-infection. (**A**) The overall clinical conditions of mice were quantitatively determined on d6 post-infection, by using a cumulative clinical scoring system (see methods) assessing the sum of the individual scores for (**B**) wasting symptoms, (**C**) diarrhea, and (**D**) fecal blood. Naive mice (open diamonds) constituted untreated and uninfected controls. Numbers of included mice are given in parentheses. Black bars indicate medians. Significance levels (*p* values) were determined with data pooled from four independent experiments using the Kruskal–Wallis test and Dunn’s post-correction.

We further quantitatively assessed the clinical outcome in *C. jejuni* infected mice upon cumin-EO treatment. Whereas at day 6 p.i., mice from the placebo group suffered from key signs of acute campylobacteriosis, such as wasting symptoms and bloody diarrhea, cumin-EO treated mice were far less clinically compromised and presented with less severe and less frequent diarrheal and wasting symptoms (*p* < 0.05–0.001 versus placebo; Figure 3). Hence, despite comparable large intestinal pathogen loads at day 6 p.i., cumin-EO treatment could alleviate macroscopic (i.e., clinical) signs of acute campylobacteriosis.

### 3.3. Microscopic Inflammatory Changes in the Colon Following Cumin-EO Treatment of C. jejuni Infected IL-10^-/-^ Mice

**Figure 4:**
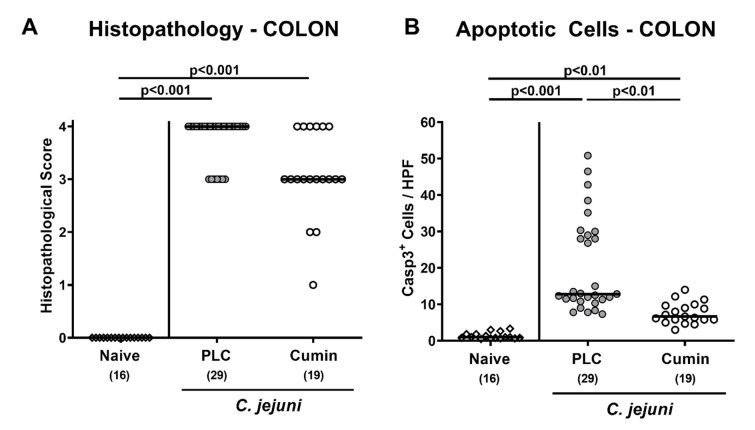
Histopathological changes and apoptotic epithelial cell responses in the large intestinal tract upon cumin-EO treatment of *C. jejuni* infected IL-10^-/-^ mice. *C. jejuni* infected secondary abiotic IL-10^-/-^ mice received either placebo (PLC, grey circles) or cumin-EO (white circles) from d2 until d6 post-infection. (**A**) Histopathological changes in H&E stained colonic paraffin sections (standardized scores) and (**B**) apoptotic epithelial cell numbers were assessed microscopically by counting cells positive for cleaved caspase-3 (Casp3^+^) from six high power fields (HPF, 400-fold magnification) per animal, respectively (see methods). Naive mice (open diamonds) constituted untreated and uninfected controls. Numbers of included mice are given in parentheses. Black bars indicate medians. Significance levels (*p* values) were determined with data pooled from four independent experiments using the Kruskal–Wallis test and Dunn’s post-correction.

We next assessed whether cumin-EO treatment resulted also in dampened microscopic inflammatory sequelae in the infected intestines. Therefore, we quantitated microscopic inflammatory changes in large intestinal paraffin sections by histopathological scoring. *C. jejuni* infection was associated with increased scores in both cohorts as compared to naive control mice (*p* < 0.001), whereas, however, a trend towards less severe histopathological changes could be observed in cumin-EO versus placebo mice (n.s., high standard deviation in the former; Figure 4A; Supplemental Figure S1A). Since apoptosis is regarded as a reliable marker for the intestinal inflammatory grading [16], we further assessed microscopic inflammatory changes in the large intestinal tract. Therefore, we quantitated numbers of apoptotic colonic epithelial cells that were positive for cleaved caspase-3 as assessed by immunohistochemical staining of large intestinal paraffin sections. *C. jejuni* infected mice exhibited markedly increased numbers of cleaved caspase-3^+^ cells in their colonic epithelia versus naive controls (*p* < 0.01–0.001) but with approximately 50% lower median numbers following cumin-EO as compared to placebo treatment (*p* < 0.01; Figure 4B; Supplemental Figure S1B). Hence, cumin-EO treatment decreased enteropathogen-induced apoptotic cell responses in the colon.

### 3.4. Immune Cell Responses in the Colon Following Cumin-EO Treatment of C. jejuni Infected IL-10^-/-^ Mice

**Figure 5:**
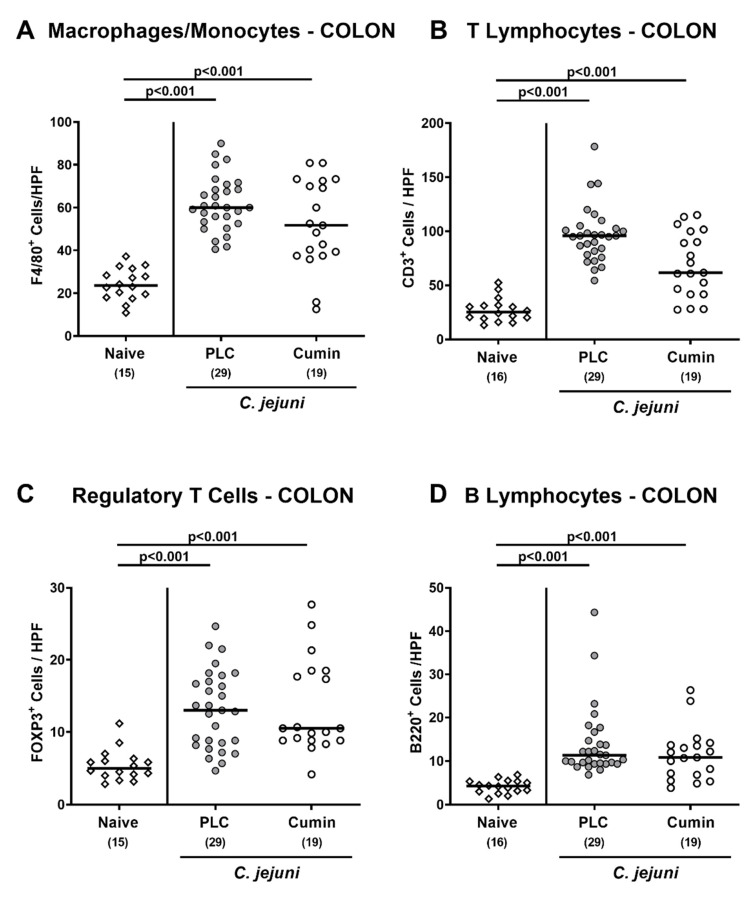
Effects of cumin-EO treatment on colonic immune cell populations in *C. jejuni* infected IL-10^-/-^ mice. *C. jejuni* infected secondary abiotic IL-10^-/-^ mice received either placebo (PLC, grey circles) or cumin-EO (white circles) from d2 until d6 post-infection. (**A**) Macrophages/monocytes (F4/80^+^), (**B**) T lymphocytes (CD3^+^), (**C**) regulatory T cells (FOXP3^+^), and (**D**) B lymphocytes (B220^+^) per mouse were determined in immunohistochemically stained colonic paraffin sections (median counts from six high power fields (HPF), 400-fold magnification) on d6. Naive mice (open diamonds) constituted untreated and uninfected controls. Numbers of included mice (in parentheses) are given together with medians (black bars). Significance levels (*p* values) were determined with data pooled from four independent experiments using the one-way ANOVA with Tukey post-correction for normally distributed data or the Kruskal–Wallis test with Dunn’s post-correction for not normally distributed data.

We further addressed whether cumin-EO application to *C. jejuni* infected mice interfered with host immune responses in the colon. Therefore, large intestinal paraffin sections were stained with antibodies against defined innate and adaptive immune cell populations and the numbers of positively stained cells within the colon mucosa and lamina propria were enumerated. In mice from either treatment group, elevated numbers of innate macrophages and monocytes, as well as of adaptive T lymphocytes, regulatory T cells, and B lymphocytes, were determined (*p* < 0.001 versus naive) but with no differences between both treatment regimens (n.s.; Figure 5). However, a trend toward lower T cell numbers (i.e., approximately 50% differences in median counts) could be assessed in the large intestines of cumin-EO compared to placebo treated mice at day 6 p.i. but did not reach statistical significance given high standard deviations (n.s.; Figure 5B). Hence, cumin-EO did not affect pathogen-induced innate and adaptive immune cell accumulation in the infected large intestines.

### 3.5. Pro-inflammatory Mediator Secretion by Mesenteric Lymph Nodes Following Cumin-EO Treatment of C. jejuni Infected IL-10^-/-^ Mice

**Figure 6:**
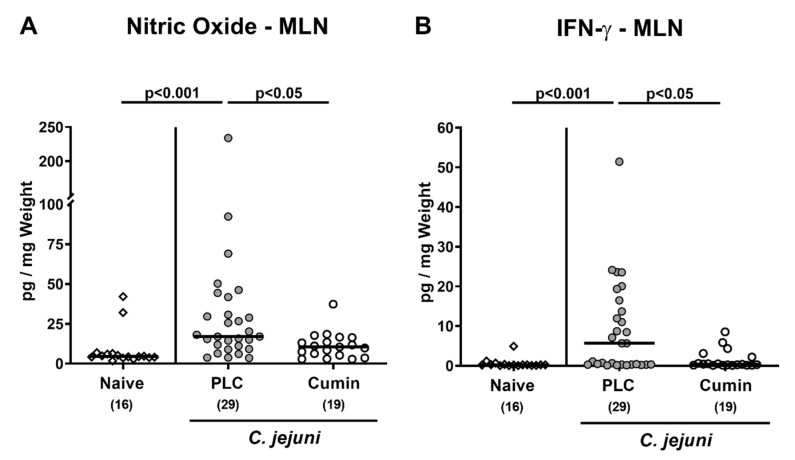
Cumin-EO treatment dampens pro-inflammatory mediator secretion in mesenteric lymph nodes of *C. jejuni* infected IL-10^-/-^ mice. *C. jejuni* infected secondary abiotic IL-10^-/-^ mice received either placebo (PLC, grey circles) or cumin-EO (white circles) from d2 until d6 post-infection. (**A**) Nitric oxide and (**B**) IFN-γ concentrations were measured in mesenteric lymph nodes (MLN) on d6. Naive mice constituted untreated and uninfected controls (open diamonds). Numbers of included mice are given in parentheses. Black bars indicate medians. Significance levels (*p* values) were determined with data pooled from four independent experiments using the Kruskal-Wallis test and Dunn’s post-correction.

We then assessed pro-inflammatory mediator secretion by MLN draining the infected intestines. On day 6 p.i., increased nitric oxide and IFN-γ concentrations could be measured in MLN derived from mice of the placebo cohort (*p* < 0.001 versus naive) as opposed to the cumin-EO group (*p* < 0.05 versus placebo; Figure 6). Hence, cumin-EO treatment decreased pathogen-induced pro-inflammatory mediator secretion by MLN back to basal levels.

### 3.6. Systemic Pro-inflammatory Mediator Secretion Following Cumin-EO Treatment of C. jejuni Infected IL-10^-/-^ Mice

**Figure 7:**
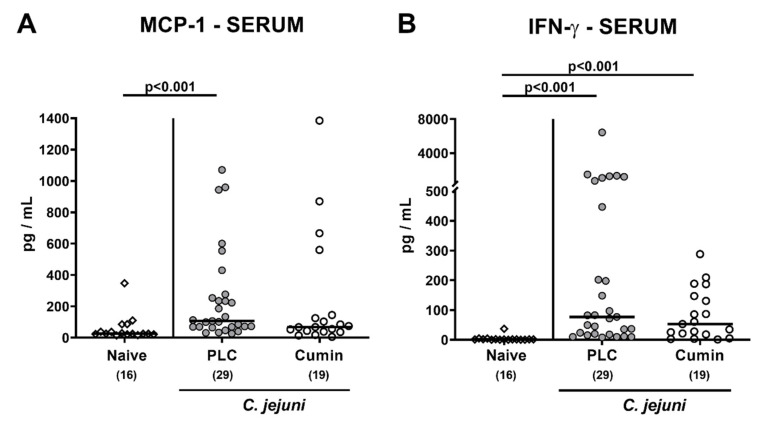
Cumin-EO treatment reduces pro-inflammatory mediators in the serum of *C. jejuni* infected IL-10^-/-^ mice. Secondary abiotic IL-10^-/-^ mice perorally infected with *C. jejuni* strain 81–176 on day (d) 0 and d1 received either placebo (PLC, grey circles) or cumin-EO (white circles) from d2 until d6 post-infection via the drinking water. On d6 post-infection, (**A**) MCP-1 and (**B**) IFN-γ concentrations were measured in serum samples. Naive mice (open diamonds) constituted untreated and uninfected controls. Numbers of included mice are given in parentheses. Black bars indicate medians. Significance levels (*p* values) were determined with data pooled from four independent experiments using the Kruskal–Wallis test and Dunn’s post-correction.

We finally addressed whether cumin-EO application might dampen pathogen-induced systemic pro-inflammatory responses by measuring distinct mediators in serum samples. Whereas mice from the placebo, but not the cumin-EO cohort, presented with increased MCP-1 serum concentrations (*p* < 0.001versus naive; Figure 7A), systemic IFN-γ secretion was comparably enhanced on day 6 following *C. jejuni* infection of mice from either cohort (*p* < 0.001 versus naive; Figure 7B). Hence, cumin-EO treatment could prevent mice from *C. jejuni* induced enhanced systemic MCP-1 secretion.

## 4. Discussion

For the first time, our here presented preclinical intervention study provides evidence for immune-modulatory effects of cumin-EO during experimental campylobacteriosis. The applied cumin concentration in the drinking water (1 g/L) applied to *C. jejuni* strain 81–176 infected mice was higher than the minimal inhibitory concentration (MIC) against *C. jejuni* strain ATCC033660, as assessed in a recent report (i.e., 0.005 g/L [37]). However, one might expect that the biologically active cumin-EO concentration within the intestinal lumen post-ingestion would be much lower given mixing and diluting with the secretory intestinal fluids. It is, thus, almost impossible to extrapolate to a MIC of the cumin-EO achieved in the intestinal lumen directed against the infecting bacteria. Furthermore, Monteiro-Neto et al. [45] investigated the oral bioavailability of cumin-EO and its compounds, such as cuminaldehyde. The results revealed that cumin-EO is a good candidate for an orally bioavailable drug and is expected to be absorbed by the gastrointestinal tract. Doses higher than 1320 mg/kg body weight would be lethal to mammalians [45]. Hence, the daily dose used in our study (i.e., 200 mg/kg body weight) was far lower than the determined potentially lethal dose. Following the therapeutic application of cumin-EO through the drinking water starting 48 h after infection, the relatively high pathogen loads of 10^9^ CFU per g feces did not change until the end of the observation period. Upon sacrifice, however, in the terminal ileum (but not in other parts of the gastrointestinal tract) of cumin-EO treated mice, lower *C. jejuni* numbers could be assessed compared to placebo controls. This result might appear rather surprising given that cumin-EO exerted not only potent antibacterial properties against *Staphylococcus* species and *Klebsiella pneumoniae* [34,35,36] but also against *C. jejuni* [37]. One needs to take into consideration, however, that these results were derived from in vitro investigations. Additionally, although cuminaldehyde is the major compound of cumin-EO, the origin of the EO itself is crucial for its composition and biological effects [46]. This could also explain the reported differences in antimicrobial activities of cumin-EO. Given the lower pathogens numbers in the terminal ileum but not the large intestine of cumin-EO as compared to placebo treated mice, it is tempting to speculate that most of the compound has been reabsorbed in the terminal ileum, whereas the concentrations of cumin-EO in the colonic lumen could have been too low for biologically relevant anti-*C. jejuni* effects. We would further like to emphasize that the 4-day cumin-EO treatment period was relatively short. Therefore, in order to achieve more pronounced benefits of cumin-EO application, a prophylactic treatment regimen starting prior infection could be advantageous and will be addressed in our future studies.

Despite the high gastrointestinal pathogen burdens, cumin-EO treated mice suffered less distinctly from clinical signs of acute campylobacteriosis as indicated by less pronounced diarrheal and wasting symptoms. These results are in line with a previous in vivo study showing that the single-dose administration of cumin extracts (500 mg/kg body weight) alleviated castor-oil-induced diarrhea in rats as indicated by lower fecal weight, less frequent defecation, and extended defecation times [32].

The better clinical outcome in *C. jejuni* infected IL-10^-/-^ mice upon cumin-EO treatment was accompanied by less pronounced apoptosis of colonic epithelial cells, whereas only a trend towards lower histopathological scores could be observed. In support, cumin treated rats suffering from gentamicin-induced nephrotoxicity showed alleviated histopathological including apoptotic changes. Furthermore, the anti-apoptotic properties of cumin could be shown in vitro, given that the Iranian cumin-EO application to murine adrenal phaeochromocytoma cells inhibited the α-synuclein fibrillation, which is known to induce apoptosis, a key pathogenic event in Parkinson’s disease [47].

When assessing potential immune-modulatory effects of cumin-EO in our acute campylobacteriosis model, we were rather surprised that numbers of innate as well as of adaptive immune cell populations within the large intestinal tract did not differ between the verum and placebo groups on day 6 p.i., as assessed by quantitative in situ immunohistochemistry. Given approximately 50% differences in median CD3^+^ cell numbers in the colonic mucosa and lamina propria, at least for the large intestinal T lymphocytes, however, a trend towards lower counts could be assessed upon cumin-EO as compared to placebo treatment on day 6 p.i. Interestingly, Chauhan et al. [48] demonstrated that oral application of cumin extracts to Swiss albino rats with cyclosporin-A induced immune-suppression resulted in elevated systemic abundance of CD4^+^ and CD8^+^ T cells, whereas the CD19^+^ B cell population was unaffected, resulting in enhanced systemic IFN-γ secretion [48]. Conversely, our study revealed that the oral cumin-EO application to *C. jejuni*-infected IL-10^-/-^ mice led to a less pronounced secretion of IFN-γ concentrations by MLN but did not affect systemic IFN-γ as opposed to MCP-1 concentrations, given that the latter pro-inflammatory mediator was increased in infected mice from the placebo but not the cumin EO cohort. These results indicate that cumin may exert very distinct immune-modulatory effects depending on its composition/formulation (given the differences observed upon application of the extract versus the EO) and the respective disease model. We could also show that cumin-EO reduced not only the IFN-γ, but also the nitric oxide concentrations in MLN derived from infected mice back to basal levels. Our results are well in line with a previous study showing that cumin-EO inhibited nitric oxide production in LPS-stimulated murine peritoneal macrophages [49]. In support, Wei and colleagues demonstrated that application of cumin-EO to LPS-stimulated murine RAW macrophages inhibited the inducible nitric oxide synthase (iNOS) and cyclo-oxygenase that was accompanied by reduced IL-6 and IL-1β expression levels upon blockage of nuclear factor-kappa B (NF-κB), the phosphorylation of extracellular signal regulated kinase and c-Jun N-terminal kinase pathways [50]. Since the transcription factor NF-κB promotes the expression of genes involved in cellular immune and inflammatory responses during early *C. jejuni* infection [51], blockage of respective pathway by cumin-EO might explain the disease-alleviating effects observed in our study. It is of note that cumin-EO is much more tolerable, given fewer undesired side effects compared to clove-EO, which was shown to be similarly effective in acute murine campylobacteriosis, as shown recently [24].

Furthermore, since it has been suggested that many essential oils and their compounds interact with the bacterial cell membrane [52], another potential strategy to combat the infection or even antimicrobial resistance is to apply non-antibiotic compounds, such as cumin-EO, in order to improve the activity of distinct antibiotic compounds. It has been shown, for instance, that cumin enhances the bioavailability of erythromycin, cephalexin, and amoxycillin [35,53]. Interestingly, α-pinene (another compound of cumin-EO) increased the sensitivity of *C. jejuni* to ciprofloxacin and erythromycin by blocking the expression of the efflux pumps encoding genes [54]. Additionally, the increase of extracellular ATP concentrations when *C. jejuni* was exposed to cumin-EO indicates that cumin demolished the membrane integrity of *C. jejuni* and permeabilized the bacterial membrane, which could result in bacterial cell death [37,55]. However, more in vivo studies are needed to further investigate the underlying mechanism of antimicrobial and immune-modulatory properties of cumin-EO during enteropathogenic, including *C. jejuni* infections, in more detail.

## 5. Conclusions

We conclude that cumin-EO is a promising immune-modulatory treatment option of acute campylobacteriosis. Additionally, the application of cumin EO as food supplementation might be a useful strategy to improve host defense mechanisms, which are involved in combating enteropathogens, including *C. jejuni*.

## Data Availability

The corresponding author provides the data from this study on request.

## Supplementary Materials

The following are available online at https://www.mdpi.com/article/10.3390/pathogens10070818/s1, Table S1: Antibiotic cocktail; Table S2: Clinical scores; Table S3: Histopathological scores; Table S4: In situ immunohistochemistry; Figure S1: Representative photomicrographs.
