# Supplementary material for: Immune-Modulatory Effects upon Oral Application of Cumin-Essential-Oil to Mice Suffering from Acute Campylobacteriosis
Source: Pathogens. 2021 Jun 29;10(7):818. doi: 10.3390/pathogens10070818 (PMC8308722; doi:10.3390/pathogens10070818)
Supplement: Supplementary file 1 [file pathogens-10-00818-s001.zip › Suppl FigS1_PICS_CUMIN-EO_COLON_28.04.21.pdf]

A

Histopathology - COLON

Naive **Placebo** **Cumin-EO**

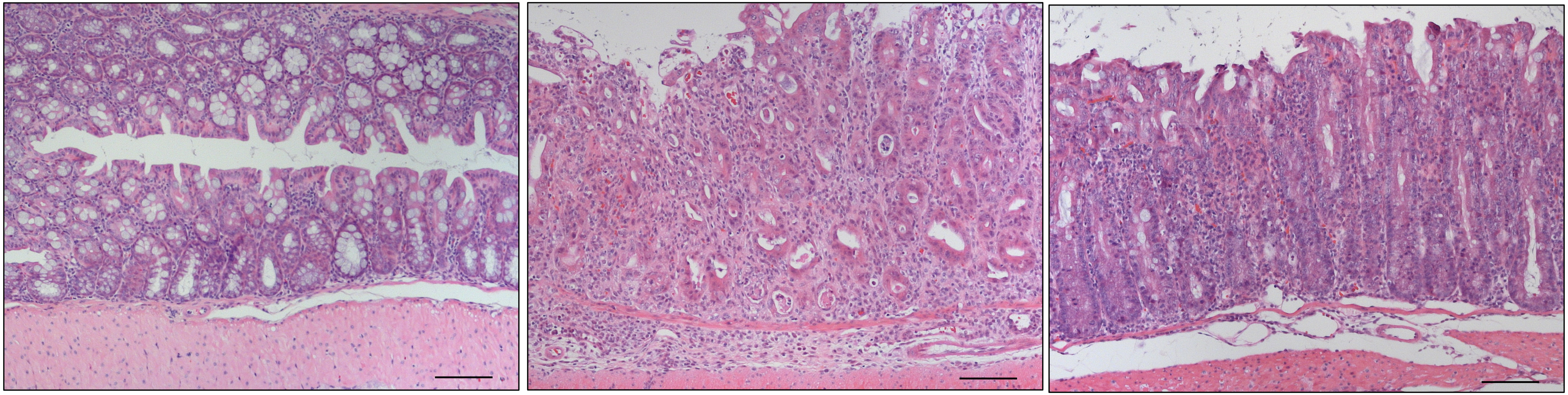

(100 x magnification, scale bar 100 µm)

B Apoptotic Cells - COLON

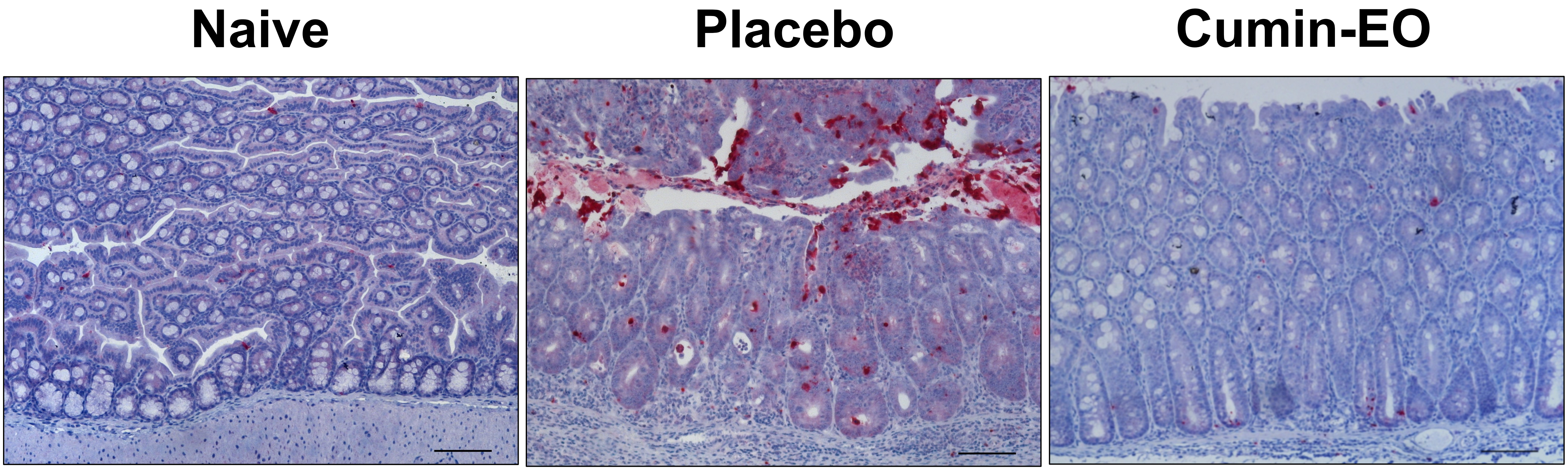

(100 x magnification, scale bar 100 μm)
