# Supplementary material for: Immune-Modulatory Effects upon Oral Application of Cumin-Essential-Oil to Mice Suffering from Acute Campylobacteriosis
Source: Pathogens. 2021 Jun 29;10(7):818. doi: 10.3390/pathogens10070818 (PMC8308722; doi:10.3390/pathogens10070818)
Supplement: Supplementary file 1 [file pathogens-10-00818-s001.zip › Supplementary_Tables1-4.pdf]

Supplementary Table S1: Antibiotic cocktail.

Antibiotic	Concentration	Company
Ampicillin plus Sulbactam	1 g/L	Dr. Friedrich Eberth Arzneimittel, Ursensollen, Germany
Vancomycin	500 mg/L	Hikma Pharmaceuticals, London, UK
Ciprofloxacin	200 mg/L	Fresenius Kabi, Bad Homburg, Germany
Imipenem	250 mg/L	Fresenius Kabi
Metronidazole	1 g/L	B. Braun, Melsungen, Germany

Supplementary Table S2: Clinical scores (maximum 12 points).

Clinical aspect	Scores
Wasting symptoms	0: normal<br>1: ruffled fur<br>2: less locomotion<br>3: isolation<br>4: severely compromised locomotion, pre-final aspect
Stool consistency	0: formed feces<br>2: pasty feces<br>4: liquid feces
Fecal blood	0: no blood<br>2: microscopic detection of blood by the Guajac method using Haemoccult, Beckman Coulter/PCD, Germany<br>4: macroscopic blood visible

Supplementary Table S3: Histopathological scores (maximum 4 points).

Score 1	Minimal inflammatory cell infiltrates in the mucosa with intact epithelium
Score 2	Mild inflammatory cell infiltrates in the mucosa and submucosa with mild hyperplasia and mild goblet cell loss
Score 3	Moderate inflammatory cell infiltrates in the mucosa with moderate goblet cell loss
Score 4	Marked inflammatory cell infiltration into the mucosa and submucosa with marked goblet cell loss, multiple crypt abscesses, and crypt loss.

Supplementary Table S4: *In situ* immunohistochemistry (colonic paraffin sections; 5 µm).

Detection	Primary Antibodies	Dilution	Company
Apoptotic epithelial cells	cleaved caspase-3	1:200	Asp175, Cell Signaling, Beverly, MA, USA,
Macrophages/ Monocytes	F4/80	1:50	no. 14-4801, clone BM8, eBioscience, San Diego, CA, USA
T lymphocytes	CD3	1:10	no. N1580, Dako
Regulatory T cells	FOXP3	1:100	clone FJK-165, no. 14-5773, eBioscience
B lymphocytes	B220	1:200	no. 14-0452-81, eBioscience
